# Readily available drugs and other interventions to potentially improve the efficacy of immune checkpoint blockade in cancer

**DOI:** 10.3389/fimmu.2023.1281744

**Published:** 2024-01-17

**Authors:** Merissa Coleman, Sophia J. Mascialino, Anusha Panjwani, Emily Edwards, Vidula V. Sukhatme, Christina Gavegnano, Vikas P. Sukhatme

**Affiliations:** ^1^Department of Pathology and Laboratory Medicine, Emory University, Atlanta, GA, United States; ^2^Center for the Study of Human Health, Emory University, Atlanta, GA, United States; ^3^College of Pharmacy, University of Florida, Gainesville, FL, United States; ^4^Morningside Center for Innovative & Affordable Medicine, Emory University, Atlanta, GA, United States; ^5^GlobalCures, Inc, Newton, MA, United States; ^6^Rollins School of Public Health, Emory University, Atlanta, GA, United States; ^7^Department of Pharmacology and Chemical Biology, Emory University, Atlanta, GA, United States; ^8^Atlanta Veterans Affairs Medical Center, Decatur, GA, United States; ^9^Center for Bioethics, Harvard Medical School, Boston, MA, United States; ^10^Department of Medicine, Emory University, Atlanta, GA, United States; ^11^Department of Hematology and Medical Oncology, Emory University, Atlanta, GA, United States; ^12^Winship Cancer Institute, Emory University, Atlanta, GA, United States

**Keywords:** financial orphans, affordable innovation, cancer, immune checkpoint blockade, drug repurposing, combination therapy

## Abstract

To improve the efficacy of immune checkpoint inhibitors (ICIs) for cancer treatment, various strategies, including combination therapies with repurposed drugs, are being explored. Several readily available interventions with potential to enhance programmed death 1 (PD-1) blockade have been identified. However, these interventions often remain overlooked due to the lack of financial incentives for their development, making them financial orphans. This review summarizes current knowledge regarding off-label drugs, supplements, and other readily available interventions that could improve the efficacy of PD-1 blockade. The summary of each intervention includes the proposed mechanism of action for combination with checkpoint inhibitors and data from animal and human studies. Additionally, we include summaries of common interventions to be avoided by patients on PD-1 blockade. Finally, we present approaches for conducting further studies in patients, with the aim of expediting the clinical development of these interventions. We strive to increase awareness of readily available combination therapies that may advance cancer immunotherapy and help patients today.

## 1 Introduction

The advent of immune checkpoint inhibitors (ICIs) represents a major advance in cancer care over the last decade. There are three major types of ICIs: drugs that intercept the CTLA-4 axis, PD-1 axis, and LAG-3 axis. A novel ICI combination with a PD-1 inhibitor, nivolumab, and LAG-3 inhibitor, relatlimab, was recently FDA-approved (1). Here we focus only on the latter since PD-1 blockade has shown efficacy in multiple tumor types and is more widely used. Indeed, it is estimated that about 40% of metastatic cancer patients are eligible for PD-1 blockade treatment.

PD-1 also known as Programmed cell death 1, is a protein expressed on T cells that causes T cell exhaustion upon interacting with either of its two ligands PD-L1/2. Antibodies to PD-1/PD-L1 are now approved for clinical use in the treatment of numerous tumor types both in the metastatic and more recently in the neoadjuvant/adjuvant setting. Predictive markers include tumor mutation burden, PD-L1 expression and mismatch repair deficiency. The overall response rate is in the 15-40% range.

**Figure 1:**
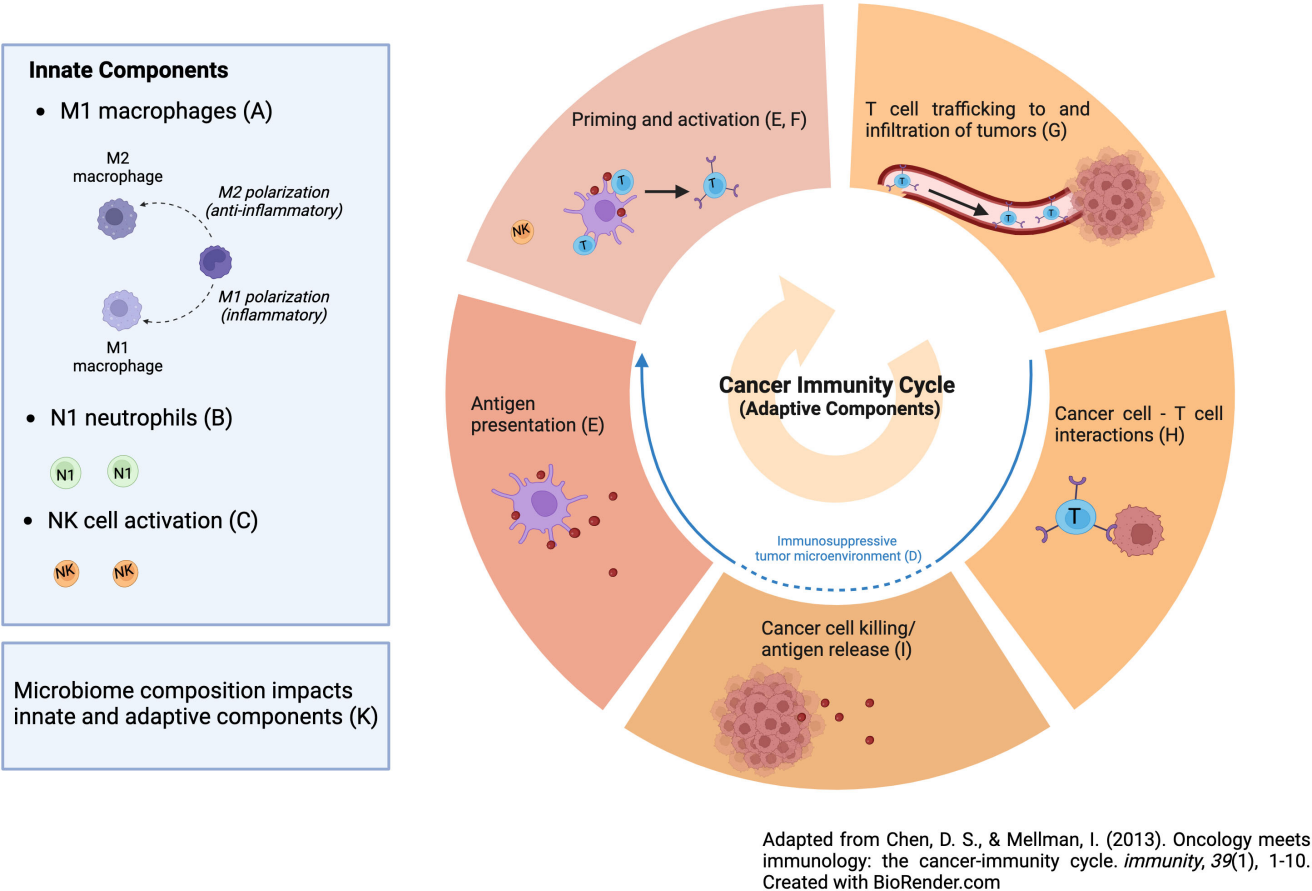
Schematic of cancer immunity cycle components needed for optimal immune-based therapies.

An area of active research is to define mechanisms of PD-1 resistance and pharmaceutical research has centered on novel therapeutics to overcome them. One way to think about resistance mechanisms to PD-1 blockade is to define the steps needed for immunotherapy to be successful, noting that countering T cell exhaustion is just one step (2). In particular, the most effective anti-tumor immune response will encompass both innate and adaptive elements as follows: M1 macrophages, N1 neutrophils, NK cell activation (innate elements), reversal of tumor-induced immunosuppression, dendritic cell activation, activation of a T-cell response, T-cell trafficking into tumor tissue, persistence of an activated T-cell response at the tumor site, T-cell engagement with tumor cells and tumor cell kill, and creation of a memory T-cell response (adaptive response, as seen in **Figure 1**) (2). Thus, PD-1 blockade is only one step in this cascade i.e., persistence of a T cell response. While pharmaceutical companies are developing new drugs aimed at improving the efficacy of ICIs by addressing the other steps, we should not ignore promising interventions that are available now that in fact do just this. Unfortunately, many of these interventions are what we have referred to as financial orphans (3), ideas that are not being aggressively pursued by pharma due to lack of a sufficient financial incentive. Here we list these opportunities and suggest ways of accelerating their clinical development. Moreover, we only cite financial orphans that are not typically considered as anti-cancer interventions. These interventions also satisfy the following two criteria: (a) there is animal data with PD-1/PD-L1 Ab and (b) human data with PD-1/PD-L1 Ab use in the form of retrospective analysis, case reports/series, and/or phase I/II trials. We conclude with interventions that might be best *avoided* if on PD-1 blockade for which there is limited human and preclinical data.

## 2 Interventions that might enhance ICI efficacy

### 2.1 Histamine 1 receptor blockers

Histamine 1 receptor (HRH1) blockers are drugs commonly used for the treatment of allergies, including allergic rhinitis and chronic idiopathic urticaria (4). These include loratadine (Claritin), desloratadine (Clarinex), diphenhydramine (Benadryl), and fexofenadine (Allegra). The binding of histamine to histamine 1 receptors triggers the release of cytokines and other immune modulators, leading to inflammation and vasodilation to optimize the removal of the allergen. HRH1 antagonists prevent histamine from binding to histamine receptors, stopping the immune cascade from creating an allergic response and the resulting subsequent symptoms.

Histamine has been implicated in the growth of cancer. Elevated levels of histamine in cancer patients have been associated with creating a more favorable tumor microenvironment (TME) for tumor proliferation (5, 6). Furthermore, mast cells, a major source of histamine, are commonly found in tumors. The binding of histamine to HRH1 receptors triggers pro-angiogenic activity, hypothesized to supply nutrients for the malignancy to grow. Additionally, the binding of histamine to macrophages results in an M2 immunosuppressive phenotype through the promotion of the immune checkpoint VISTA, diminishing CD8+ action against tumor cells (6). Thus, this group hypothesized that HRH1 blockers might provide a synergistic effect when used with PD-1 blockade.

Histamine levels while mice underwent PD-1/PD-L1 treatment were investigated. It was found that in mice with EO771 tumors treated with PD-1/PD-L1 Ab, mice with weak responses to the treatment had higher HRH1 expression and VISTA pathway activation because of H1 binding. Mice treated with the HRH1 blocker fexofenadine along with PD-1/PD-L1 blockade had prolonged survival. Of mice treated with PD-1 Ab and fexofenadine, 50% remained tumor-free compared to only 10% of mice treated only with PD-1 Ab. Additionally, mice treated with PD-1 blockade and fexofenadine were observed to have higher CD8+ activity.

This group also examined the effects on cancer survival of histamine blockers in humans in a retrospective cohort study. Researchers included a total of 3,544 cancer patients, 878 with melanoma, 342 with breast cancer, 1,937 with lung cancer, and 387 with colon cancer. All patients were receiving cancer treatment, including immune checkpoint blockade therapy (PD-1/PD-L1 immunotherapy), or chemotherapy. Of the 40 common drugs taken among cancer patients, only HRH1 blockers were significantly associated with improved patient survival (P=0.005), and this association was only demonstrated in patients taking immunotherapy. Compared to age, sex, and/or stage-matched patients who did not take HRH1 blockers, patients taking HRH1 blockers were noted to have a statistically significant reduction in death. The most significant reductions in death rates were observed in melanoma and lung cancer. Decreases were also observed in breast and colon patients; however, there were not enough patients in these groups for statistically significant results. It was also found the HRH1 activation increased T cell dysfunction in 9 of 12 cancer types evaluated. Low numbers of CD8+ T cells were observed in the tumor, while histamine and HRH1 were upregulated in the tumor microenvironment (TME). Of note, tumor response in 70 patients with multiple solid tumor types correlated inversely with pretreatment histamine levels, especially above 0.6 ng/ml. Collectively this data suggests that H1 blockade might be most efficacious in those patients with “high” level expression of HRH1 on tumor associated macrophages and high (approximately > 0.6 ng/ml) histamine blood levels.

Similarly, a recent study investigated the use of six common antihistamines in a retrospective cohort study of Swedish cancer patients (n=429,198) with immunogenic and non-immunogenic tumors (7). It was found that the HRH1 blocker, desloratadine demonstrated the greatest reduction in hazard ratio of all HRH1 blockers compared to cancer patients who took no supplemental drugs in addition to their cancer therapy. Desloratadine showed the most significant effect on immunogenic cancers, including gastric, colorectal, anal, pancreatic, lung, breast, prostate, kidney, bladder, melanoma, and Hodgkin’s lymphoma. The most statistically significant decrease in hazard ratio was observed in melanoma with a HR of 0.50 [95% Cl (.32-.78)]. Breast, prostate, and pancreatic cancers supplemented with desloratadine all demonstrated significant reductions in hazard ratio at 0.75 [95% Cl (0.61-0.92)], 0.75 [95% Cl (0.61-.93)], and 0.71 [95% Cl (0.56-0.90)] respectively.

### 2.2 Beta blocker (propranolol)

Beta-blockers (BBs) inhibit beta-adrenergic receptors that help transduce the action of the catecholamines norepinephrine and epinephrine. Commonly used for cardiovascular diseases, BBs have been evaluated for cancer treatment where they act antagonistically on receptors that stimulate tumorigenesis and tumor metastasis (8). Propranolol is a nonselective BB that binds to beta-1/beta-2 specific receptor subtypes. It is the most used beta-blocker for cancer indications due to its low toxicity profile and data showing the anti-cancer effects are mediated by the beta-2 receptor (8).

Combined treatment with propranolol and anti-PD-1 blockade (RMP1-14) is associated with decreased PD-1 expression in CD8+ TILS and decreased tumor growth in the 4T1 murine tumor model (9). Interestingly, in this model, either intervention alone showed little to no efficacy. In another study using the B16-F10 aggressive melanoma murine model, treated with anti-PD1 Ab, propranolol, and IL-2 interventions, the most statistically significant delay in tumor growth was in animals administered the anti-PD1 Ab/propranolol combination (10). The combination of propranolol and IL-2 also resulted in a significant delay in tumor growth, making it a potential combinatory treatment. In these murine models, PFS and OS increased but no disease regression was observed. BBs do not act directly on the PD-1/PD-L1 pathway, but may be modulating the tumor microenvironment, decreasing expression of PD-1 and improving progression-free survival (9). Treatment of propranolol in MT/ret murine models reduced myeloid infiltrate by 49% and significantly inhibited polymorphonuclear neutrophils (PMNs) as well as increased natural killer (NK) cells and CD8+ cytotoxic T cells (11).

In a retrospective study in patients with metastatic melanoma (n=195), those who received pan-BBs experienced a significant survival benefit compared to patients taking no beta-blocker or a B1-selective blocker (10). In a small retrospective study (n=109), patients taking beta-blockers and PD-1 inhibitor experienced significant PFS increase [HR = 0.58, 95% CI: 0.36 – 0.93] compared to those not taking beta-blockers (12). At Roswell Park, clinicians conducted a phase I 3 + 3 dose escalation study for metastatic melanoma patients receiving propranolol and pembrolizumab (13). Seven of the nine patients experienced no TRAE above grade 3 and the study concluded that because frequency of adverse events was not higher than anti-PD1 therapy alone, combinatory treatment was safe. There was also preliminary evidence of efficacy with 7/9 patients showing objective responses.

### 2.3 Flu vaccine

The seasonal influenza vaccine is currently the most efficacious intervention for prevention and control of the influenza virus. The vaccine targets strains of hemagglutinin (HA) and neuraminidase (NA) antigens of the influenza A & B viruses. It has been shown to stimulate tumor CD-8+ T cells and decrease regulatory B cells (14). The vaccine acts as a catalyst for stimulating antitumor immune responses, converting immunologically cold tumors to “hot” (14). These immune mediated responses increase the efficacy of ICIs such as PD-1. In an animal study analyzing the combination of an unadjuvanted seasonal influenza vaccine (FluVx) and anti-PD-1 Abs, tumor growth reductions surpassed those noted with either intervention alone (14).

In a multi-center retrospective cohort study (n=303), improved PFS and OS, was noted in the vaccination group (15). In particular, multivariate PFS analysis showed that influenza vaccination showed better PFS [HR = 0.63, 95% CI: 0.41–0.98, *p*-value = 0.041] “after adjustment for age, gender, CCI, performance status, CNS metastasis, and line of treatment”. Similarly, in multivariate analysis (adjusted for age, gender, CCI, performance status, CNS metastasis, line of treatment) for OS, the study found a statistically significant difference of OS in favor of the vaccinated group [HR = 0.53, 95% CI = 0.30–0.93, *p*-value = 0.028]”.

One note of caution, there is mixed toxicity data in humans when the influenza vaccine is given in combination with PD-1 inhibitors. A retrospective cohort study (n=23) conducted in Switzerland found that 52.2% of patients experienced immune-related adverse effects (IRAE) including encephalitis and autoimmune peripheral neuropathy (16). This study set off a series of alarms, causing additional studies to be conducted on the relationship between IRAE and the combinatory intervention, none of which supported this study. Moreover, in the multi-center retrospective cohort study noted above, the IRAE incidence was similar between both groups (15). In another retrospective cohort study (n=370) in patients with non-small cell lung cancer (NSCLC), no difference in IRAE was found either (17). A randomized controlled study done at the Mayo Clinic (n=108) saw no significant difference in adverse effects in patients receiving vaccination with the checkpoint inhibitor therapy (18). Thus, it is unclear why the study in Switzerland found such conflicting results, but small sample size may be a possibility.

### 2.4 L-Arginine

L-Arginine is an amino acid used for nitric oxide production by endothelial and immune cells (19). Supplementation confers benefits for reducing blood pressure in hypertensive adults including pregnant women with gestational hypertension (19). Down regulation of argininosuccinate synthetase (ASS), which synthesizes arginine from citrulline, is associated with cancers including melanoma, hepatocellular carcinoma, prostate carcinoma, lung & colon carcinomas, sarcomas, invasive breast carcinoma, and renal cell carcinoma (20–22). Furthermore, an increase in L-arginine levels induces a shift from glycolysis to oxidative phosphorylation in T cells and promotes memory cells in a murine model (23). In another *in-vivo* study examining only L-arginine, the treatment inhibited myeloid-derived suppressor cells (MDSCs) and increased concentrations of CD4+ and CD8+ T cells (24). These animal results suggest that supplementation may improve T-cell survival and may act synergistically with the PD-L1 pathway to elicit antitumor effects.

In immunocompetent mice models with osteosarcoma, increased OS was found with combined L-arginine and anti-PD-L1 antibody treatment (25). The combined treatment showed survival at 79.5 days as compared to anti-PD-1 antibody treatment alone at 39 days. L-arginine supplementation alone did not show improved survival. The two interventions worked synergistically, with L-arginine supplementation increasing the number and activity of CD8+ T-cells and the antibody protecting the cells from immune exhaustion. This study showed inhibition of tumor growth, but no evidence of disease regression.

In a randomized controlled trial (RCT) of patients with various advanced cancers (n=296), low arginine levels at baseline (<42 uM) were significantly associated with worse OS [median OS = 38.8 months vs. 24.6 months; HR = 1.57; 95% CI: 1.10 – 2.24; *P* = 0.012] and associated with high PD-L1 expression in cells, indicating that a combinatory therapeutic approach may yield favorable outcomes (26). Potential biomarkers are CD4+ and cytotoxic CD8+ cells, as not all studies have found reduction in myeloid-derived suppressor cells (MSDCs).

### 2.5 Fenofibrates

Fenofibrates are FDA approved therapeutics for the management of hyperlipidemia and high cholesterol. They regulate PPAR-a and Wnt pathways to increase beta-oxidation and reduce LDL-C (27, 28). In HPV+ head and neck squamous cell carcinoma (HNSCC), fenofibrate use was effective and added to the efficacy of cisplatin, the most common chemotherapeutic for HPV+ HNSCC (29). Fenofibrates led to a decrease in PD-L1 expression in hypoxic conditions. Given that PD-L1 suppresses immune function, fenofibrates effectively reprogram the tumor microenvironment. In an additional study, researchers found that because CD8+ tumor infiltrating lymphocytes (TILs) are linked to fatty acid catabolism, fenofibrates increased T cell function and promoted TIL concentrations (30). Investigators in both studies deduced that utilization of fenofibrate may exhibit synergistic effects in conjunction with anti-PD1 antibodies.

Bezafibrates, which promote fatty acid metabolism and lower cholesterol, have also been evaluated in conjunction with PD-1 blockade (31). These therapeutics mechanistically activate PPAR-a as well as PGC-1 to stimulate fatty acid oxidation. Bezafibrates alone did not exhibit anti-tumor efficacy but in conjunction with anti-PD-L1 Ab, they increased cytotoxic T lymphocyte concentrations and promoted their survival by modulating metabolic processes.

In a cohort study, veterans (n=3593) with NSCLC treated with a checkpoint inhibitor, either an anti-PD1 or anti-PDL1 antibody, were evaluated for fibrate exposure (32). Researchers described a “modest increase in OS” for patients that received the combinatory therapeutic. Of note, fibrates did not improve OS in those receiving chemotherapy. These findings imply that fibrates in isolation do not manifest intrinsic antitumor effects; instead, they augment responses to immunotherapy.

### 2.6 Metformin

Metformin is a widely prescribed anti-hyperglycemic oral medication used to manage type 2 diabetes and polycystic ovary syndrome. The exact mechanisms of metformin action have been uncertain since its discovery in the 1920s (33, 34). Metformin may have effects on other bodily processes, including activation of AMP-activated protein kinase (AMPK) through inhibition of mitochondrial respiratory chain complex 1 (35). AMPK is a key energetic sensor essential in controlling T-cell metabolic reprogramming that is also involved in differentiation and specification of T-cell activity (36). Thus, given the role of AMPK in enabling the proliferation of anti-tumor immunity via T cell differentiation, it is hypothesized that metformin may synergize with immunotherapy, particularly the PD-1 blockade.

The impact of metformin and anti-PD-1 immunotherapy on colon cancer and melanoma *in vitro* and *in vivo* mouse models has been investigated (37). In mice with colon cancer, 88% of mice who received combined therapy demonstrated complete tumor regression compared to the expected 40% clearance by anti-PD-1 Ab alone. Similarly, combined therapy in murine melanoma models resulted in tumor regression in 80% of mice and complete regression in 70% of mice. Mechanistically, the researchers demonstrated that metformin reduced hypoxia in the TME via inhibition of tumor oxygen consumption. T-cell oxygen consumption increased, likely due to the higher availability of oxygen with lower tumor cell oxygen consumption. While the study did not mention the increased reactive oxygen species (ROS) generated through inhibition of mitochondrial respiratory chain complex 1, it is possible that increased ROS as a byproduct of metformin allowed additional anti-tumor T cell activity while preventing tumor consumption of oxygen. However, despite the strikingly high rates of tumor regression observed in melanoma mouse models with anti-PD-1 and metformin, combination therapy was only effective for very small tumors.

In humans, small studies have been unable to determine with confidence the impact of combined immunotherapy and metformin on clinical outcomes. A retrospective study of melanoma patients (n=34) who took metformin while receiving either nivolumab or pembrolizumab anti-PD-1 treatment found no change in progression-free survival or overall survival compared to patients receiving anti-PD-1 alone (38). However, another study analyzed combinational metformin and immunotherapy, including anti-PD-1 and CTLA4, in advanced melanoma patients (n=55) and found improved clinical outcomes compared to immunotherapy alone (39). The median overall survival in the combinational therapy group was 46.7 months compared to 28 months. Additionally, by the third-year follow-up, 73.3% of patients who received combined therapy were still alive compared to only 20.7% of patients who received immunotherapy alone. Median progression free-survival in the combinational therapy group was calculated at 19.8 months compared to 5 months in the immunotherapy-only group. Despite these improved outcomes, cohort differences in overall survival nor progression-free survival were deemed statistically significant. The authors point to the small sample size as a likely contributor to statistical insignificance.

### 2.7 Statins

Statins lower the amount of low-density lipoprotein cholesterol, often termed “bad cholesterol”, present in the body (40). Specifically, statin inhibits the conversion of HMG-CoA to mevalonate, greatly reducing the amount of cholesterol produced and in turn lowering risk for cardiovascular disease. Further, by reducing the level of HMG-CoA, statins disrupt the first step of the isoprenoid biosynthetic pathway which in turns leads to a decrease in the levels of isoprenoids farnesyl pyrophosphate (FPP) and geranylgeranyl pyrophosphate (GGPP) (41). By disrupting this pathway and causing a reduction in both FPP and GGPP, statin promotes tumor-specific apoptosis, providing evidence for its use in clinical settings.

Because intracellular cholesterol is important in maintaining PD-1 levels in cells, statins can be used to decrease PD-1 expression. Previous studies have revealed the importance of AKT and B-catenin pathways in mediating intracellular cholesterol levels (42). In lung cancer and melanoma cells, statins have been shown to inhibit both the AKT and B-catenin pathways, therefore decreasing the level of PD-1 expression. This provides evidence for statin’s use as a cancer therapeutic and has been used to inform further studies in animals and humans (42).

The effects of statins on *KRAS* tumors in CD-1 mice has been tested (43). Results showed that statins promoted immunogenic cell death (ICD) of *KRASmut* cancer cells and led to an increase in the CD8+ T-cell immune response against *KRASmut* tumors. Furthermore, a meta-analysis of 13 studies (n=3331) investigated the effect of various NSAIDs in combination with PD-1 Ab on PFS and OS (44). Five of these studies focused on statins, and the researchers found that patients on statins with NSCLC and malignant pleural mesothelioma (MPM) treated with PD-1 blockade showed improvement in progression free survival and overall survival, compared to non-statin users [OS: HR 0.76, 95% CI (0.63–0.92), *P* = .005; PFS: HR 0.86, 95% CI (0.75–0.99), *P* = .036)] (44).

### 2.8 Aspirin/NSAIDs

Aspirin is non-steroid anti-inflammatory drug (NSAID) that is often used to treat pain and fever. Aspirin and other NSAIDs inhibit cyclooxygenases (COXs) that lead to the production of prostaglandins (PGs) and thromboxanes, which lead to inflammation. Aspirin can suppress PD-1 and PD-L1 signaling in ovarian cancer cells (45). Lysine acetyltransferase 5 (KAT5), an acetylase that has been shown to induce the acetylation of both histone and non-histone proteins, has been shown to be a potential target for cancer treatments. Specifically, aspirin works by suppressing KAT5 which has been shown to inhibit PD-1/PD-L1 signaling. Aspirin enhanced the effect of anti-PD-L1 therapy in *in vitro* tumor models (45). PGE2 elevation is associated with poorer prognosis in PD-1 treated tumors (44). Mechanistically, this link may be driven by the cDC1-dependent checkpoint, which is modulated by NSAIDs.

A meta-analysis investigating 13 studies on ICI and NSAID combination therapy, five of which focused on aspirin, found that patients with MPM and NSCLC who were treated with both PD-1/PD-L1 inhibitors and aspirin showed improved progression free survival but not overall survival [low-dose aspirin users *versus* non-aspirin users: OS: HR 0.93, 95% CI (0.76–1.15), *P* = .514; PFS: HR 0.84, 95% CI (0.72–0.98), *P* = .024] (44). These results provide evidence for the potential to use aspirin in combination with PD-1/PD-L1 inhibitors to suppress tumor growth and improve progression free survival.

Further, diclofenac, another NSAID, has been shown to have unique effects on tumor progression in combination with anti PD-1. Not only does diclofenac improve the killing of T cells *in vitro* in combination with anti-PD1, it also inhibits lactate transporters, specifically monocarboxylate transporter 1 and 4 (46). This leads to a decrease in the outflow of lactate from cells, which in turn minimizes the lactate secretion of tumor cells. Further, diclofenac, in combination with ICIs, has been shown to have significant implications in humans diagnosed with NSCLC (47). A retrospective study in patients with NSCLC (n=3634) in which 2,336 patients received both ICIs and were exposed to NSAIDS has been conducted. Results of this study revealed that NSAIDs were associated with improved overall survival [HR 0.90, 95% CI (0.83-0.98), *P* = .010). Specifically, diclofenac was found to be the only NSAID associated with significant overall survival in addition to having the lowest hazard ratio [HR 0.75, 95% CI (0.68-0.83), *P* <.001). These studies done *in vitro* and in humans reveal the promise that aspirin, diclofenac, and other NSAIDs may reduce tumor growth when combined with PD-1 checkpoint inhibitors.

### 2.9 Angiotensin receptor

Angiotensin Receptor Blockers (ARBs) are a class of drugs often used to treat hypertension, heart failure, and chronic kidney disease. Although they can be used to target many different parts of the renin-angiotensin pathway, they ultimately result in lowering blood pressure and inducing protective cardiovascular and renal effects (48). Since the renin-angiotensin system is involved in the regulation of cell proliferation, it has been hypothesized that ARBs may have potential to be used in regulating tumor growth. From an immune perspective, Angiotensin Receptor Blockers are thought to be involved in the down regulation of transforming growth factor (TGF), a protein that is intimately tied to immunosuppression potentially through induction of Treg cells (49). Previous research has shown that patients with metastatic urothelial carcinoma (mUC) who received PD-1/PD-L1 inhibitors showed increased TGF levels in the blood. Therefore, a study sought to investigate whether combination of ARBs and PD-1/PD-L1 inhibitors would result in increased tumor suppression growth in patients with mUC. This retrospective cohort study (n=178) revealed that combination of angiotensin receptor blockers with PD-1/PD-L1 inhibitors resulted in further suppression of tumor growth in patients with metastatic urothelial carcinoma than when they were just treated with PD-1/PD-L1 inhibitors. Additionally, it has been found that some ARBs are peroxisome proliferator-activated receptor- γ (PPAR- γ) and increase PD-1 blockade as well as cytotoxic T lymphocyte‐mediated antitumor activity (50, 51). A retrospective study (n=167) was conducted to assess the impact of combining PPAR- γ activating ARBs and PD-1 blockade on survival of patients with different types of cancer was conducted (52). Compared to patients who were only treated with PD-1 blockade, patients who also received PPAR- γ activating ARBs showed both improved progression free survival and overall survival and had about a 50% reduction in mortality rates and disease progression. This study provides further evidence for the use of ARBs in combination with anti PD-1 as a means for improving survival rates in cancer patients.

### 2.10 Vitamin B5

Vitamin B5 serves as the precursor to coenzyme A and is present in many food items that humans consume daily. Recent studies in mice have shown that vitamin B5 and coenzyme A promotes mitochondrial metabolism and help CD8+ T cells differentiate into IL-22 producing Tc22 cells, a population of tumor cytotoxic cells (53, 54). Additionally, vitamin B5 supplementation has been correlated with increased response to PD-1 therapies and Coenzyme A enhanced the activity of T cells *in vitro* in mice. When testing the impact of vitamin B5 on mice who were also given a PD-L1 specific antibody, vitamin B5 enhanced the response of MC38 tumors to the antibody (53).

In a small subset of human patients, plasma levels of vitamin B5 were positively correlated with response to PD-1 targeted therapies (53). It was found that found that melanoma patients who responded to PD-1 had higher levels of B5 in their plasma than patients who did not respond to PD-1, providing further evidence of the impact that Vitamin B5 has in improving response to PD-1 immunotherapies (54). Collectively, there is sufficient evidence to suggest that combining vitamin B5 and PD-1/PD-L1 therapies has the potential to suppress tumor growth.

### 2.11 Magnesium

Magnesium participates in numerous biochemical reactions. Its impact on cellular immunology and on cancer has been investigated recently. It was found that magnesium is necessary for activating the opening of LFA-1, an integrin on CD8+ T cells (55). This finding indicates magnesium’s key role in modulating T cell function and response and suggests that it might synergize with ICIs.

A study in mice to investigate whether a combination of magnesium and PD-1 blockade could inhibit tumor growth has been conducted (55). Administration of magnesium suppressed MC38 tumor growth in mice. The researchers then tested whether a combination of magnesium and PD-1 blockade could improve tumor-directed memory CD8+ T cell function. This combination induced the strongest CD8+ T cell response, providing evidence for the use of magnesium in combination with PD-1 blockade in regulating tumor growth.

Importantly, low magnesium levels correlated with distinctly worse outcomes in CAR-T cell therapy for B cell lymphoma and in PD-L1 Ab treatment for NSCLC.

### 2.12 Manganese

Manganese is an essential nutrient for cellular function. Delivered as MnCl_2_, Mn^2+^ is FDA approved to be administered orally, intramuscularly, and intravenously (56). The effects of Mn^2+^ on immunity have been elucidated recently. One mechanism of the innate immune system is driven by the STING ligand which activates and recruits cytotoxic T cells to sites of cytosolic DNA that can indicate the presence of cancer cells (57). Mn^2+^ may be integral in activating the cGAS-STING pathway by increasing cGAS reactivity to cytosolic DNA, thereby inducing a more potent immune response including cytotoxic T cells (56). STING-deficient mice lack CD8+ mobilization in response to cancer therapies, indicating the importance of the STING pathway in CD8+ biology (56). Moreover, deficient Mn^2+^ levels resulted in higher susceptibility to tumor occurrence in mice injected with melanoma cells. Additionally, mice with colon adenocarcinoma or melanoma administered supplemental Mn^2+^ had lower tumor burdens than mice who did not receive Mn^2+^ injections and elevated CD8+ activity in tumors. When coupled with anti-PD-1 treatment, mice who received Mn^2+^ supplementation exhibited reduced tumor growth compared to those only receiving immunotherapy. However, it is important to note that this effect was more significant in the melanoma mouse model compared to the colon adenocarcinoma model. It was hypothesized that dendritic cell maturation was induced by Mn^2+^ which enabled the relatively low immunogenicity of B16 melanoma mouse model to trigger a stronger immune response than usually observed in this model.

A phase I clinical trial patients evaluating patients with metastatic cancer (n=22) has been conducted (56). Patients received intranasal Mn^2+^ with dose escalation in addition to anti-PD-1 immunotherapy and chemotherapy. MnCl_2_ was either administered intranasally or by inhalation at 0.5 or 0.1 mg/kg or 0.1, 0.2 or 0.4 mg/kg, respectively, once a day for three weeks. Of the patients enrolled, five patients who had previously failed to respond to the combination of immunotherapy and chemotherapy or radiation responded to the Mn^2+^, immunotherapy, and chemotherapy combination. Two patients were able to maintain stable disease while three patients demonstrated partial response. While treatment adverse events were observed in 86% of patients ranging from mild to serious, all adverse events resolved. Furthermore, it is likely that these adverse events were related to the immunotherapy and chemotherapy combination as no Mn^2+^ overdose toxicities were observed.

### 2.13 Fecal microbiota transplant

The gut microbiota consists of various bacteria and microorganisms that line the GI tract (58). In addition to benefitting the body in several other ways, the gut microbiota plays a vital role in the immune system, protecting the body against pathogens and helping to build immunity by impacting the development of both the innate and adaptive immune system (58, 59). Consequently, disruptions in the gut microbiota are thought to underlie many immune-mediated disorders. Further, specific compositions of the microbiota may be better suited to fight pathogens and respond to immunotherapies, and the specific bacteria present in the microbiota may influence the degree to which a patient responds to antitumor immunotherapies.

A preclinical study done in mice with melanoma found that differences in the bacteria present in the intestinal microbiota led to differences in antitumor immunity (60). Further, the presence of bacteria *Bifidobacterium* specifically improved the regulation of tumor growth to the same degree as PD-1/PD-L1 therapies had. It has been proposed that *Bifidobacterium* increased dendritic cell function which caused an increase in the CD8+ T cell accumulation in the tumor microenvironment. A study in humans building off of this produced similar results, providing evidence for the manipulation of the gut microbiota in congruence with cancer immunotherapies (61). Melanoma patients who responded to PD-1 immunotherapy (n=30) had more favorable gut microbiomes, including a higher diversity and abundance of bacteria in the Ruminococcaceae family (62). Additional studies in humans found that patients with abnormal microbiomes due to use of antibiotics exhibited resistance to ICIs that targeted the PD-1/PD-L1 pathway (63). Further, a study done in human melanoma patients (n=128) revealed that a high fiber diet was associated with improved progression free survival in patients receiving immune checkpoint blockade. This study was partnered with a preclinical study which revealed that a low fiber diet or taking a probiotic were each associated with a lower response to anti-PD-1 therapies as well as a lower frequency of cytotoxic T cells in the tumor microenvironment (64). The probiotic data is worth noting since a particular probiotic (Clostridium butryicum – see below) has beneficial effects. A high fiber diet in the microbiome triggered intratumural type 1 interferon (IFN-1) and natural killer T cell (NK)- dendritic cell cross talk. Activation of this pathway improved response to immune checkpoint blockade, revealing how the microbiome influences the tumor microenvironment (65).

These three studies provide evidence for the use of Fecal Microbiota Transplantation (FMT) as a cancer immunotherapy. An FMT involves the transfer of fecal matter from a donor with a desirable microbiota to a recipient in order to change and benefit the recipient’s microbiota (66). Multiple phase 1 human clinical trials have revealed that the combination of fecal microbiota transplant and PD-1 inhibitors can successfully change the gut microbiota and overcome resistance to anti-PD-1 treatment (67–69). A fecal microbiota transplant from a cancerous mouse who responded to ICI treatment into an antibiotic treated mouse increased response to ICI treatments, whereas the reverse transplant did not (63). Further, in a human phase 1 clinical trial with melanoma patients (n=10), three of the patients treated with FMT showed desirable changes in gene expression in the tumor microenvironment and in another human phase 1 clinical trial, a subset of melanoma patients (n=6) revealed that a combination of FMT and anti-PD-1 contributed to a change in the microbiota and a reprogramming of the tumor environment which allowed for the overcoming of resistance to anti-PD-1 (67). In another phase I RCT (n=29), a PD-1 inhibitor (nivolumab) and CTLA-4 inhibitor (ipilimumab) were assessed with usage of Clostridium butyricum (fortified yogurt) (70). In the combinatory treatment, 74% of patients saw a reduction in tumor target lesions in addition to increased PFS and OS. Concerning safety profile, grade 3 and 4 toxicities were observed including “fatigue, rash, adrenal insufficiency, hyperglycemia, and diarrhea”; two patients were discontinued from the treatment but resolved with corticosteroid therapy. Collectively, these studies provide evidence for the safe and effective use of fecal microbiota transplants to regulate tumor growth and increase response to ICI treatments such as PD-1/PD-L1 blockades.

## 3 Interventions and circumstances to consider avoiding with ICI administration

### 3.1 Acetaminophen

Acetaminophen is a widely used FDA approved drug for pain and fever relief though the mechanism remains elusive. It may achieve its effects by crossing the blood-brain barrier and acting on the central serotonergic system (71). However, this view is contested by other assertions that acetaminophen blocks cyclooxygenase (COX) enzymes implicated in the formation of prostaglandins, molecules that control inflammation and pain. Although patients often use acetaminophen to relieve cancer treatment-induced pain, acetaminophen has been implicated in dampening the immune response, including the immunotherapy-induced immune response. While the exact mechanism of immunosuppression is unknown, several studies have suggested that acetaminophen should not be taken during immunotherapy.

In mice with colon tumors, mice treated with safe doses of acetaminophen and anti-PD-1/anti-PD-L1 had increased regulatory T cells in their tumors and lower overall survival than mice receiving anti-PD-1/anti-PD-L1 alone (72). The increased tumor-infiltrating regulatory T cells suggest that acetaminophen may have an immunosuppressive effect that inhibits immune activation by immunotherapy. It should be noted that acetaminophen increased CD68+ macrophage tumor infiltration and led to decreased tumor size in *athymic* rats, thus extrapolation to the immune-competent setting may be problematic (73).

In a cohort study of patients (n=297) with various solid tumor types receiving nivolumab, an anti-PD-1 antibody, patients who had detectable levels of acetaminophen in their plasma had worse progression-free survival and overall survival (72). Patients with detectible acetaminophen had a median progression-free survival of 2.63 months and median overall survival of 8.43 months. Comparatively, patients without detectible plasma acetaminophen had a median progression-free survival of 5.03 months and median overall survival of 14.93 months [95% CI (0.53-0.91), *P*=0.009; 95% CI (0.32-0.69), *P* < 0.0001].

In patients (n=225) who received pembrolizumab as either a first- or second-line therapy, it was found patients who took a high dose of acetaminophen (exceeding 24 hours of intake or exceeding 60 doses of 1000 mg) within 30 days before, during, or 90 days after treatment had significantly worse outcomes in for patients undergoing either first- or second-line therapy (74). High doses of acetaminophen showed independent impacts on PFS and OS on multivariate analysis. Notably, low doses of acetaminophen (not exceeding 24 hours of intake or not exceeding 60 doses of 1000 mg) were not associated with significant decreases in PFS or OS. This data alludes to a dose-dependent impact, suggesting that low doses may be permissible, but patients should avoid acetaminophen in general, especially high doses.

### 3.2 Proton pump inhibitors

Gastric hydrogen potassium ATPase (HK-ATPase) is an integral membrane protein found in cells that line the stomach. These proteins are responsible for pumping gastric acid into the stomach. However, over-production of stomach acid and improper closing of the esophagus can lead to painful conditions, such as gastroesophageal reflux disease, peptic ulcers, and Barrett’s esophagus (75). Most proton pump inhibitors are competitive HK-ATPase inhibitors which inhibit the release of stomach acid.

There is a growing body of evidence suggesting that PPIs may deter the therapeutic effect of immunotherapy due to their indirect effects on the gut microbiota, a key regulator of the immune response. The use of PPIs was associated with decreased gut microbiota richness, a key indicator of gut health (76). The 221 patients on PPIs out of the 1,815 patients sampled demonstrated overgrowth of certain bacterial species that indicated increased susceptibility to infection. Particularly, overgrowth of genera *Enterococcus*, *Streptococcus, Staphylococcus*, and *Escherichia coli* were observed, all of which are associated with increased risk of invasive *C. difficile* infections. Rich and diverse gut microflora have been linked to improved immunotherapy outcomes in patients receiving anti-PD-1 immunotherapy (61). Thus, homogeneity of the gut microbiota promoted by PPIs may weaken immune activation via immunotherapy, worsening clinical outcomes.

In a retrospective study of patients (n=227) with urothelial carcinoma treated with a PD-1 inhibitor, it was found patients who took PPIs during treatment had significantly worse clinical outcomes compared to patients who did not take PPIs (61). The median immune progression free survival (iPFS) was 2.5 months in PPI users compared to 4.1 months in non-PPI users (p=0.00014). More notably, OS was reduced from a median of 18.8 months in non-PPI users to 9.5 months in PPI inhibitor users (p=0.00026). It was also observed that the negative effects of PPIs on clinical outcomes were less significant as age increased but more significant in men than women. These parameters are of interest to consider when assessing patient-specific risk in a situation where immunotherapy and PPIs need to be administered together.

Similarly, a larger retrospective study of patients (n=802) with urothelial carcinoma across 22 counties found decreased PFS and OS in patients taking PPIs while receiving pembrolizumab treatment compared to patients not taking PPIs [PFS 4.5 vs 7.2, p=0.002; OS 8.7 vs 14.1, p=0.001] (77).

Another study observed similar detrimental effects on overall survival in patients (n=118) who had taken PPIs within the 60 days of beginning immunotherapy as the aforementioned studies [HR 2.47, 95% CI (1.28-4.74) P = .007] (78). One study retrospectively investigated both the individual and dual usage of PPIs and antibiotics in patients (n=212) receiving anti-PD-1 immunotherapy in various solid cancers (79). It was found that patients who took PPIs during treatment had lower overall response, progression-free survival, and overall survival than patients who did not take PPIs. The hazard ratios of PPI PFS and OS were 1.51 [95% CI (1.11-2.05), *P* < 0.001] and 1.89 [95%Cl (1.23-2.90), *P* = 0.002], respectively. Notably, the largest risk to PFS and OS was observed in patients who took both antibiotics and PPIs with hazard ratios of 3.65 [95%CI (2.75-4.84), *P* < 0.001] and 2.12 [95%Cl (1.37-3.27), *P* = 0.002].

While it may be advised to stop PPI usage before and during immunotherapy, patients may find reducing or avoiding PPI usage difficult. In another retrospective multivariable analysis of NSCLC patients (n=425), a history of PPIs was independently associated with shorter PFS survival when compared to ICI monotherapy in patients without PPI usage [HR 1.38, 95% CI (1.00-1.91), *P*=0.048] (80). Interestingly, patients with a history of PPI usage who received both ICIs and chemotherapy had similar outcomes to patients who did not take PPIs. These findings suggest that the deleterious effects of PPI usage on immunotherapy is mitigated by additional treatment types, such as chemotherapy (80). However, chemotherapy in addition to ICIs may not be suitable for all patients. Administering the probiotic Clostridium butyricum along with the PPI may be another alternative. A retrospective analysis of 16S fecal genome sequencing in 118 non-small cell lung cancer patients indicated that, due to the microbiota-driven effects of PPIs on immunotherapy efficacy, using this probiotic may neutralize the deleterious effects of PPIs (78). Notably, compared to patients who only took PPIs during immunotherapy, patients who had taken both PPIs and Clostridium butyricum had a higher OS of NR compared to 208 days [HR 0.42, 95% CI (0.19-0.92), *P* = .030]. Similarly, patients who had taken both PPIs and Clostridium butyricum demonstrated improved PFS of 250 days compared to only 88 days in PPI and immunotherapy-only patients [HR 0.52, 95% CI (0.29-0.94), *P* = .030]. The observed improvements to OS and PFS were sustained at 3-years post-treatment, indicating long-term counteractive effects of probiotic usage on PPI hazard during immunotherapy treatment. Furthermore, the guts of patients who took Clostridium butyricum had less colonization with disease-associated bacteria, such as *Atopobium* and *Streptococcus*, underlying the role of the gut microbiome. Research into mitigating these effects via probiotics or other gut-health-promoting mediums provides opportunities to improve therapeutic outcomes.

### 3.3 Antibiotics

Antibiotics have been integral in exterminating and preventing bacterial infections for decades since the discovery of penicillin in 1928. While bacterial infections once contributed to the bulk of disease across the globe, the introduction of antibiotics decreased overall bacterial infection burden (81). Since their discovery in 1928, several classes of antibiotics targeting various types of bacteria have been developed. Despite the rather small percentage of bacteria that cause deadly infections, most bacteria found on and inside the human body i.e., the microbiome, are crucial to human health. Recent research indicates that antibiotics and cancer therapies may be at odds with one another due to the importance of the microbiome in regulating the immune response.

The diversity of the microbiota is crucial to regulating the activity of the immune system. Antibiotics do not have the specificity to target a single bacterial species causing an infection. As a result, important bacteria that prevent the overgrowth of other more invasive bacteria are unable to have a protective effect when eliminated by antibiotics. The overgrowth of various bacterial species can result in inflammation and alter immune function (81).

A study disrupted mice microbiotas using a combination of antibiotics ampicillin, metronidazole, neomycin, and vancomycin preceding injection with colon tumor cells and subsequent infusion of anti-PD-1 mouse antibodies (82). At day 24 post-tumor-inoculation, mice who were treated with probiotic Lactobacillus rhamnosus two weeks before tumor injection had a smaller tumor size than mice treated without probiotics, 1,681.02 ± 77.86 mm^3^ compared to 2,511.05 ± 83.64 mm^3^ *(P=*0.014). Similarly, overall survival rate at day 24 was 77.8% in mice treated with the probiotic in addition to anti-PD-1 antibody compared to 33.3% in mice treated with the anti-PD-1 antibody alone. Additionally, 13 key species of bacteria were found to be affected by the initial antibiotic administration. However, at day 24, the gut microbiotas of mice treated with probiotics had been restored to pre-antibiotic conditions which was not observed in mice who did not receive probiotics. Due to the necessity of antibiotics, particularly in patients with cancer-weakened immune systems, encouraging mouse model data suggests the need for additional research into probiotics for mitigating the negative-side effects of antibiotics in humans.

With regards to human data, a study sequenced the 16S ribosomal DNA from stool samples of 38 melanoma patients before undergoing anti-PD-1 immunotherapy to investigate the effect of the gut microbiota on clinical outcomes (61). It was found that patients with more diverse gut microbiotas responded to anti-PD-1 immunotherapy while patient’s with less gut diversity were less likely to respond. Thus, antibiotics may be removing the microbial diversity essential to mediating a positive response to immunotherapy. A retrospective study followed the clinical outcomes of 142 non-small cell lung cancer patients who started anti-PD-1/PD-L1 immunotherapy between January 2016 and January 2018 (83). The researchers found reduced progression-free survival and overall survival in patients who received antibiotics 1 month before, during, or 1 month after treatment. The average progression-free survival fell from 9.63 months in patients who did not take antibiotics to 3.73 months in patients who took antibiotics (p < 0.0001). Perhaps more drastically, overall survival fell from 21.87 months to only 6.07 months in patients who received antibiotics (p < 0.0021). While the infection itself may have contributed to the decrease in overall survival in patients who received antibiotics, the compound effect of infection and antibiotics on patient outcomes suggests the importance of preventing infection and limiting antibiotic use in cancer patients receiving immunotherapy. Additional retrospective studies have found concurrent data, suggesting that antibiotics may interrupt the immunotherapy-activated anti-tumor immune response (63, 84).

Similar deleterious effects of antibiotics on immunotherapy have been observed in patients receiving PPIs, as noted above, both of which have been linked to their effects on the microbiome. Indeed, probiotics appear to counteract the microbial disruption of PPIs, restoring the effects of immunotherapy (78). There is no human data on the potential neutralizing effects of probiotics on antibiotic treated-immunotherapy patients.

Further honing in on the importance of the microbiome, a study of 16 melanoma patients who had previously failed to respond to anti-PD-1 therapy received fecal microbiota transplants (FMT) from patients with complete or partial responses to anti-PD-1 treatment. Six patients who had previously had no response to anti-PD-1 immunotherapy and had progressive disease demonstrated clinical benefit after receiving FMT (68). Objective response was observed in 3 patients and stable disease in 3 additional patients. While the patients had all received anti-PD-1 therapy previously, 6 patients failed to respond until their gut microbiota was altered by the gut microbiota of anti-PD-1 responding-patients. Moreover, genomic sequencing found that patients who responded to immunotherapy post-FMT had more significant and lasting changes to their pre-FMT bacterial gut composition. Not only does FMT offer an intriguing addition to immunotherapy but furthers the importance of preserving essential bacteria for immunotherapy response by limiting the use of antibiotics. The data also suggests that investigating the use of probiotics to improve immunotherapy efficacy by decreasing the deleterious effects of antibiotics is relevant and should be built upon.

### 3.4 Late day anti-PD-1 administration

A recent area of interest in immunology has been the effect of the circadian rhythm on the body’s ability to create a substantial immune response to various immune stimulants, including immunotherapy and vaccines. The circadian rhythm is an overarching term that encompasses the processes within the body that occur in 24-hour cycles, mainly responding to the amount of light outside. These processes regulate the release of hormones and thus cellular activity (85). While the release of melatonin in the sleep-wake cycle is widely known, other hormones and cellular processes that operate on a circadian rhythm are less well-studied.

Given that circadian rhythms affect various biological processes, recent research has attempted to investigate the effect of the circadian rhythm on the immune system. The effects of the circadian rhythm have been specifically demonstrated in T cells, which are the target of immunotherapies, such as anti-PD-1. In a study of T cell activity *in vitro* and *in vivo* in mice, it was found that when CD28, a costimulatory signal in the maturity of naïve T cells, was released at constant high rates, T cells still demonstrated differences in rates of maturity during day compared to the night (86). Additionally, the *Clock* gene generates cycles of 24-hour physiological rhythms. Mice with a knocked-out gene for the CLOCK protein failed to show differentiation in T cell proliferation during the day compared to the night which normal mice demonstrated (86). The inability for the mice without circadian rhythms to generate T cell proliferation patterns aligned with daylight, as observed in mice with the *Clock* gene, suggests that T cell activity is controlled by circadian rhythms independently of other proteins and cells.

By measuring immune cell activity, such as mobilization of B cells and T cells, it has been demonstrated that adaptive immunity, the specialized “memory” immunity, is more robust when vaccines are administered in the morning or early afternoon rather than the evening (87–89).

Given the preclinical and clinical data indicates that the immune system follows a circadian rhythm, it may be hypothesized that leveraging immune system activity may allow for immunotherapy treatments to be more effective when administered during certain times of the day. Immunotherapy is often administered monthly, so it may be argued that the body undergoes several circadian rhythms while the drug is in the body. However, pembrolizumab concentrations are highest in key anti-tumor T cell activation areas, such as lymph nodes, as little as 30 minutes after infusion (90). Thus, immunotherapies, such as anti-PD-1, may be able to reach more T cells if there is a larger quantity of active T cells when drug concentrations are highest. In a retrospective analysis of a 9-year longitudinal study, researchers collected data on 299 patients with stage IV melanoma who received infusions of pembrolizumab, ipilimumab, and nivolumab (91). The participants were grouped into those who received less than 20% of their infusions after 4:30 PM and those who received at least 20% of their infusions after 4:30 PM. It was found that latter group had a crude overall survival hazard ratio of 1.31 [95% CI (1.00-1.71), *P* =0.046]. In an attempt to control the variables between the patients in the earlier and later administration groups, a propensity score analysis including 73 patients from each group was conducted. Patients were matched by age, serum lactate dehydrogenase levels, and treatment with radiotherapy and/or corticosteroids. In terms of 5-year survival rates, propensity-matched patients with at least 20% of their administrations past 4:30 PM had 5-year survival rates of 49% compared to 68% for patients who received less than 20% of their infusions in the evening [HR 2.16, 95% CI (1.10-4.25), *P* = 0.025] (91). Propensity-matched patients showed a strong association with poorer outcomes in the later administration group, with an OS hazard ratio of 2.04 [95% CI (1.04-4.00), *P* = 0.038]The overall progression-free survival (non-propensity matched) was measured at 40% [95% CI (30–54), *P* = 0.041] for those who received at least 20% of their administrations past 4:30 PM EST compared to 56% [95% CI (50–63), *P* = 0.041] for those who received infusions earlier in the day. Thus, this data suggests the importance of timing in administering immunotherapy.

It is important to note that there were several differences between the two infusion groups. Out of the 299 total participants, only 74 patients received at least 20% of their infusions past 4:30 PM while 225 patients received less than 20% past 4:30 PM. The sample sizes in each group alone could skew the representation of patient outcomes. While the researchers used a propensity analysis of 73 patients from each group to control the imbalance, the earlier infusion group received more infusions of PD-1 Abs than the patients with later infusion times. Thus, some of the variable outcomes between the groups could be attributed to the number of treatments. Additionally, in the propensity-controlled group, more ICI administrations in the earlier infusion group occurred in the summer when there is more light and patients may be spending more time outside. Additionally, the 299-patient cohort was largely male dominated with 66% of the total participants being male and only 34% being female. While this ratio was carried throughout both infusion groups, it may not be representative of the effects of the circadian rhythm on the general population.

Additional retrospective studies on melanoma, NSCLC, squamous cell carcinoma of the esophagus, and renal cell carcinoma provide additional information on the role of the circadian rhythm in immunotherapy efficacy. Later infusion times have been associated with worse outcomes in renal cell carcinoma (92). One study on renal cell carcinoma found a continuous association with the number of treatments, finding a 16% increased risk of death for every 10% of infusions after 4:30 PM (93).

A retrospective analysis of patients (n=95) with advanced NSCLC found that patients who received anti-PD-1 antibody, nivolumab, between 9:27 AM and 12:54 PM nearly quadrupled their PFS compared to those who received nivolumab between 12:55 PM and 5:14 PM [11.3 months PFS, 95% CI (5.5-17.1), P < 0.001 compared to 3.1 months PFS 95% CI (1.5-4.6), P < 0.001] (94). A similar observation was made for OS, with OS for the morning group nearly quadruple the OS of the afternoon group [34.1 month OS, 95% CI (15.1-53.3), P < 0.001 compared to 9.6 months OS, CI 95% (4.9-14.4), P < 0.001]. Another study of patients (n=180) with NSCLC found decreases in PFS for patients who received at least 20% of immunotherapy treatment after 4:30 PM compared to those who received less than 20% of infusions after 4:30 PM (95). However, in contrast, this study did not observe a significant difference in OS [HR 1.48, 95% CI (0.99-2.20), P = 0.055]. When including the number of infusions given to patients in each treatment group as a variable in the Cox model, there were no statistically significant differences in a multivariate analysis of PFS or OS between patients who received less than 20% of their infusions after 4:30 PM and patients who received at least 20% of their infusions after 4:30 PM [PFS HR 1.20, 95% CI (0.83-1.75), P=0.329; OS HR 1.11, 95% CI (0.73-1.67), P=0.636]. Therefore, for future studies, controlling the number of infusions may be important in determining the role of the time of day.

Interestingly, the time of day may be relevant during certain stages within the patient’s immunotherapy treatment schedule. A study of patients (n=62) with squamous cell carcinoma of the esophagus found that patients who received nivolumab for the first time before 1:00 PM had better outcomes than those who received nivolumab for the first time after 1:00 PM [PFS HR 0.40, 95% CI (0.22-0.71), P=0.002; OS HR 0.49, 95% CI (0.26-0.95), P=0.036] (96). Similarly, for patients within the first 3 months of treatment with nivolumab, earlier infusion times were associated with better PFS and OS [PFS HR 0.45, 95% CI (0.26-0.79), P=0.005; OS HR 0.68, 95% CI (0.37-1.26), P=0.224]. However, the same trend was only weakly observed for all-time treatment courses [PFS HR 0.75, 95% CI (0.44-1.28), P=0.303; OS HR 0.93, 95% CI (0.50-1.72), P=0.821). This data suggests that patient outcomes may be more heavily impacted by infusion time earlier in the patient’s treatment course.

Further retrospective analysis of stage IV melanoma patients (n=73) found general trends towards improved outcomes for patients who received more than 75% of their treatments before 2:00 PM, particularly when measuring OS (97). Most of note, this study found that the most striking improvements to PFS and particularly OS were found in women, older patients, patients with fewer metastases, and less CNS involvement. Hence, certain demographics may benefit most from infusion timing.The research conducted on the effects of the circadian rhythm on the immune system and the subsequent effects on treatments that target the immune system has demonstrated a likely connection between earlier immunotherapy administration and patient outcomes. Due to the association, it may be advised for physicians to avoid giving infusions of immunotherapy past the early afternoon in the local time zone. While this may not be consistently feasible with patient scheduling, limiting the number of immunotherapy treatments a single patient receives beyond the early afternoon to less than 20% could prove beneficial for patient outcomes (91). The synergetic benefits in harnessing the body’s natural immune activity fluctuations via the circadian rhythm could confer substantial improvement in immunotherapy patient outcomes but randomized studies are called for.

## 4 Conclusion

Despite the fact that immune checkpoint blockade has had an unmistakable positive impact on the treatment of cancer, there remains substantial room for improvement. The aforementioned non-cancer drugs and interventions when given along with ICIs exhibit early but promising results in animals and humans. These results should be regarded as a springboard to encourage further human studies, especially RCTs. Furthermore, the findings in the latter part of this paper, namely interventions to avoid with ICI use, should be considered when advising patients on medications to avoid or limit during immunotherapy treatment.

Physicians may be hesitant to deviate from the prescribed anti-PD-1 infusions with the addition of another drug/supplement when there is not the highest level of supporting evidence in the form of RCTs. We recognize that RCTs are the gold standard for evaluating the efficacy of new interventions. While RCTs are an imperative next step to gain widespread acceptance and reimbursement for the interventions, there are some challenges posed by the nature of these interventions. Indeed, the very attributes that make these interventions attractive (potential efficacy, affordability, and safety profile) are ones that make them less likely to garner funding from pharmaceutical companies, since there is minimal or no financial benefit from the resulting data, unless the data clearly results in a new indication for the ICI. Indeed, pharma is more likely to pursue the next blockbuster that may synergize with immune checkpoint blockade. Thus, funding for RCTs will need to come from the government or via philanthropy. Moreover, testing multiple interventions simultaneously such as maintaining Mg and Mn levels during immunotherapy while administering an H1 blocker in those with high pre-treatment blood levels are not as practical to carry out in an RCT format.

With these facts in mind, while waiting for RCTs to start, one can argue that for the ~100,000 cancer patients who are started on ICIs annually, we should consider another approach to validating the efficacy and safety of the interventions we cite. Caregivers should be educated about these interventions and a registry created to assess outcomes, both of which we are in the process of doing. The registry will also gather real world data on ICI use and outcomes, while also providing efficacy and safety signals in patients who receive interventions mentioned in this review. Of course, the decision to treat should be conducted in a shared decision-making model between caregiver and patient with risks and benefits personalized to each case. Even after such a registry is created, we acknowledge that there will be logistical, regulatory, legal, and financial issues that need addressing for such treatments to be offered by physicians at scale outside of a clinical trial.

The research above outlines ways in which the drugs target various immunogenic tumor-proliferation techniques that may synergize with anti-PD-1 immunotherapy. While each drug in combination with immunotherapy alone has illustrated anti-cancer effects, the diverse mechanisms of action of the interventions reviewed here suggest that the best results may be observed when multiple interventions are administered together (**Table 1**). Also, though the strong safety profiles make the risk-benefit ratio for patients with late-stage, metastatic disease reasonable for physicians to consider, it remains to be seen if untoward toxicities arise when more than one of these interventions is considered along with ICI use.

**Table 1:** Summary of Interventions & Clinical Endpoints.

	Type of Evidence	Human Study End Points (PFS, OS, Hazard Ratio)	Number of Patients*	Mechanism of Action	Clinical Takeaways	Type of Cancer
Outcome Improving Interventions						
HRH1 Blocker	PC + HR	OS (6) (7) + Hazard Ratio (7)	n=3544 (6), n=429,198 (7)	A + F	Desloratadine had the highest reduction in hazard ratio ONLY when combined with PD-1 blockade and no other drugs	melanoma, breast cancer, lung cancer, colon cancer
Beta Blocker	PC + HR	PFS (9, 10, 12) + OS (10) + Hazard Ratio (12)	n =195 (10) n=109 (12), n=9 (13)	D	The use of beta blockers with PD-1 inhibitors increased PFS [HR 0.58 95% CI (0.36-93)] without conferring additional adverse events compared to immunotherapy alone (12, 13)	metastatic melanoma
Flu Vaccine	PC+ HR + RCT	PFS + OS + Hazard Ratio (15)	n=23 (16), n=303 (15), n=370 (17), n=108 (18)	E	Although one study reported patients experiencing immune-related adverse effects (IREA) with PD-1 inhibitors, later studies revealed no difference in IREA between experimental and control groups, suggesting that the use of flu vaccine is safe during immunotherapy (15–18)	NSCLC, melanoma
L-Arginine	PC + HR	OS (26)	n=296 (26)	D	High arginine levels at baseline are significantly associated with higher OS [median OS = 38.8 months vs. 24.6 months; HR = 1.57; 95% CI: 1.10 – 2.24; P = 0.012] (26)	metastatic osteosarcoma, NSCLC, HNSCC, renal cell carcinoma, colorectal, lymphoma, gastric, endometrial, cholangiocarcinoma, anal, vulvar, small cell lung carcinoma, ovarian, neuroendocrine, cervical cancer
Fenofibrates	PC + HC	OS (32)	n=3593 (32)	I	Fibrates improved OS in patients receiving PD-1 blockade, but not in those receiving chemotherapy (32)	NSCLC, HNSCC
Metformin	PC + HR	PFS + OS (39) (not statistically significant)	n=34 (38), n=55 (39)	H	PFS and OS improvement were not statistically significant (39)	melanoma, colon cancer
Statins	PC + HR	PFS + OS + Hazard Ratio (44)	n=3331 (44)	E + H	Combination of statin with PD-1 blockade showed improved progression free survival and overall survival in humans (44)	lung cancer, melanoma, NSCLC, MPM
NSAIDs (Aspirin + Diclofenac)	PC + HR	Aspirin: PFS + Hazard Ratio (44) Diclofenac: OS + Hazard Ratio (47)	n=3331 (44), n=3634 (47)	aspirin: D + E, diclofenac: D + E + I	Combination of diclofenac with PD-1 blockade showed signifcant overall survival and the lowest hazard ratio in humans (47)	NSCLC, MPM
FMT	PC + H1 + RCT	PFS + OS (70)	n=30 (62), n=10 (63), n=128 (64), n=6 (67), n=29 (70)	K	Two patients were discontinued from the study due to grades 3 and 4 toxicity level, which were resolved corticosteroid therapy (70)	melanoma
Angiotensin Receptor	PC + HR	PFS + OS (52)	n=178 (49), n = 167 (52)	D + H	Combination of PPAR- γ activating ARBs and PD-1 blockade decreased disease progression (49, 52)	urothelial carcinoma, breast, gastrointestinal, gynecological cancers, head and neck, heptobiliary, lung, pancreatic, renal, skin
Vitamin B5	PC + HR	N/A	n=42 (54)	F + I	Elevated B5 plasma levels were associated with higher rates of anti-PD-1 therapy response in cancer patients (53, 54)	melanoma
Magnesium	PC + HR	N/A	n=100, n=65 (55)	F	Combination of magnesium and anti PD-1 blockade produced the most robust CD8+ T cell response (55)	B cell lymphoma, NSCLC
Manganese	PC + HC	N/A	n=22 (56)	A + C+ E + F	Adverse events (all resolved) appeared to be related to the combination of immunotherapy with chemotherapy (56)	colon adenocarcinoma, melanoma, advanced metastatic cancer
Outcome Hindering Interventions						
Acetaminophen	PC + HR	reduced OS + PFS (72)	n=297 (72), n=225 (74)	Unknown	Patients with detectable levels of acetaminophen in their plasma had approximately half the PFS and OS compared patients without detectable levels of acetaminophen in thier plasma (72)	NSCLC, melanoma, soft-tissue sarcoma, renal cell carcinoma, urothelial carcinoma, cervix carcinoma, triple-negative breast carcinoma, gastric, head and neck, renal, colorectal cancer
Proton Pump Inhibitors	PC + HR	reduced OS + PFS (79) + increased Hazard Ratio (80)	n=227 (61), n=1815 (76) n=802 (77), n=118 (78), n=212 (79)	K	The combination of PPIs and antibiotics showed the worst hazard ratio (79)	urothelial carcinoma, melanoma, NSCLC, renal cell carcinoma, head and neck cancer
Antibiotics	PC + HR	reduced OS + PFS (83)	n=38 (61), n=142 (83)	K	Probiotics appeared to reverse effects on the gut microbiota and restore the response to immunotherapies (78) (79)	melanoma, NSCLC
Late Day Anti-PD-1 Administration	HR	reduced OS + PFS + increased Hazard Ratio (91)	n=299 (91), n=145 (92), n=104 (93), n=95 (94), n=180 (95), n=62 (96), n=73 (97)	Unknown	Increased hazard ratio when patients received treatment after 4:30pm (91)	melanoma, NSCLC, renal cell carcinoma, squamous cell carcinoma

Color Scheme: light purple= interventions that might enhance ICI efficacy. light yellow= interventions that might hinder ICI efficacy.

Type of Evidence: HR, human retrospective; HC, (human) case report or series; H1, phase 1 data; H2, phase 2 data; PC, preclinical data; RCT, randomized controlled trial.

Human Study Endpoints: OS, Overall Survival; PFS, Progression Free Survival.

Number of Patients: *Total number of patients enrolled in each retrospective cohort, case report, or randomized control trial, including patients who both received PD-1 blockade and those who did not.

Mechanism of Action: A) M1 macrophages B) N1 neutrophils C) NK cell activation D) reversal of tumor-induced immunosuppression E) dendritic cell activation F) T-cell activation G) T-cell trafficking into tumortissue H) prevention of T-cell exhaustion and/or optimization of T-cell metabolism I) T-cell engagement with tumor cells and tumor cell kill J) creation of a memory T-cell response K) microbiome composition.

Type of Cancer: NSCLC, non-small cell lung carcinoma; MPM, malignant pleural mesothelioma; HNSCC, head and neck squamous cell carcinoma.

With the burden of cancer on the healthcare system, and more importantly on human lives, the anticipation for a blockbuster cancer drug is eagerly awaited. However, there are low-cost, widely available interventions backed by promising evidence that could be deployed immediately in patients receiving ICIs in the hope of improving patient outcomes. We suggest that both randomized clinical studies and off-trial treatments coupled to a registry for outcomes evaluation be given immediate and serious consideration.

## Author contributions

MC: Writing – original draft, Writing – review & editing. SM: Writing – original draft, Writing – review & editing. AP: Writing – original draft, Writing – review & editing. EE: Visualization, Writing – original draft, Writing – review & editing. VS: Conceptualization, Writing – review & editing. CG: Funding acquisition, Writing – review & editing. VS: Conceptualization, Funding acquisition, Writing – original draft, Writing – review & editing.
